# A Hybrid Approach to Model Reduction of Generalized Langevin Dynamics

**DOI:** 10.1007/s10955-025-03404-1

**Published:** 2025-01-28

**Authors:** Matteo Colangeli, Manh Hong Duong, Adrian Muntean

**Affiliations:** 1 Department of Information Engineering, Computer Science and Mathematics, University of L’Aquila, L’Aquila, Italy; 2 School of Mathematics, University of Birmingham, Birmingham, UK; 3 Department of Mathematics and Computer Science & Centre for Societal Risk Research (CSR), Karlstad University, Karlstad, Sweden

**Keywords:** Generalized Langevin dynamics, Model reduction, Invariant manifold method, Fluctuation–dissipation theorem

## Abstract

We consider a classical model of non-equilibrium statistical mechanics accounting for non-Markovian effects, which is referred to as the Generalized Langevin Equation in the literature. We derive reduced Markovian descriptions obtained through the neglection of inertial terms and/or heat bath variables. The adopted reduction scheme relies on the framework of the Invariant Manifold method, which allows to retain the slow degrees of freedom from a multiscale dynamical system. Our approach is also rooted on the Fluctuation–Dissipation Theorem, which helps preserve the proper dissipative structure of the reduced dynamics. We highlight the appropriate time scalings introduced within our procedure, and also prove the commutativity of selected reduction paths.

## Introduction

Complex systems, commonly encountered in the study of physical and biological processes as well as in social sciences (e.g. in molecular dynamics, motion of human crowds or fish swarms, opinion formation in political elections, etc.), are often described by coupled systems of stochastic differential equations (SDEs). These are meant to model the time evolution of a large number of microscopic constituents interacting with one another, possibly also coupled to some external reservoirs. The stochastic character of the equations is introduced to account for a certain degree of uncertainty in the description of the phenomenon—which is intrinsic in many applications, and may originate from various sources, e.g. noisy and rapidly changing environments—as well as the presence of unavoidable human estimate errors [4]. The noise term thus allows to meaningfully encode the details of the microscopic interactions with certain unknown factors (unresolved particles or reservoirs) endowed with prescribed statistical properties. This approach turns out to be of vital importance in the analysis of stochastic climate models, see e.g. the seminal paper by Hasselmann [28] and the recent review [40].

However, the resulting SDEs are often hard to treat analytically, and even their numerical characterization may not be easily accessible, typically owing to the high dimensionality of the model (large number of degrees of freedom, or of the involved parameters, etc.). It is thus of both theoretical and practical relevance to describe large complex systems through simpler and lower dimensional ones, while still preserving the essential details of the original models. Over the last two decades, such research endeavour has gradually developed and systematized into the growing field of model reduction methods, which are now applied not only to SDEs but also to other types of mathematical models, such as ODEs and PDEs [3, 19, 22, 43, 49, 51].

A natural and largely studied reduction approach consists in projecting the full Markovian dynamics onto a manifold parametrized by a set of relevant, typically slow, variables (for instance, certain marginal coordinates), sometimes called “collective variables” in the literature [27]. However, within this approach, a non-Markovian dynamics, characterized by the presence of some memory terms, is typically obtained [56]. It is then desirable to restore the Markovian structure of the original process, so as to avoid the onset of memory terms in the dynamics. In the set-up of fast–slow systems, which constitute the focus of most existing works in the literature, as the ratio of fast-to-slow characteristic time-scales increases, the fast dynamics equilibrates more and more rapidly with respect to the slow ones; consequently, a fully Markovian reduced description for the evolution of the slow variables can be derived in the limit of a perfect time-scale separation, see e.g. [24, 56] and the monographs [22, 46]. More recently, there has been an increasing interest towards the development of techniques of model reduction not relying on the aforementioned separation [48]. In [53, 54] the authors present a reduction procedure, based on the Edgeworth expansion, for both deterministic and stochastic dynamics displaying a moderate time-scale separation. In [7, 52] the Ruelle–Pollicott resonances are investigated in a reduced state space in which time-scales are only weakly separated. The papers [15, 16, 26, 33, 35, 37, 38, 55] deal, instead, with diffusion processes for which no time-scale separation is explicitly invoked, advocating an approach based on conditional expectations.

In this paper we apply a reduction procedure to the generalized Langevin dynamics, which is a fundamental model in nonequilibrium statistical mechanics. Our goal is essentially twofold. On the one hand, we aim to recover and connect, within a common theoretical framework, different Langevin equations widely known and debated in the literature, describing underdamped or overdamped dynamics, and sometimes also including heat bath variables. On the other hand, we also want to highlight meaningful corrections to the foregoing equations, originating from the lack of a perfect time-scale separation. The work is organized as follows. In Sect. 2 we introduce the Generalized Langevin Equation and discuss the various reduction paths examined throughout the manuscript. In Secs. 3 we study the derivation of the underdamped Langevin equation from the Generalized one, whereas in Sect. 4 we pursue the reduction procedure further in order to obtain the overdamped Langevin from the underdamped one. In Sect. 5 the overdamped equation is instead obtained directly from the Generalized one, by simultaneously removing the momentum and the heat bath variables. Next, in Sect. 6, we derive an overdamped equation for a system coupled to a set of heat bath variables. The heat baths are finally erased from the latter description, in Sect. 7, so that the standard overdamped equation is obtained through a suitable time scaling. Conclusions are drawn in Sect. 8.

## The Generalized Langevin Equation

The Generalized Langevin Equation (GLE) is given by [5, 45]

1a $$\begin{aligned} dq&=\frac{p}{m}\, dt,\end{aligned}$$

1b $$\begin{aligned} dp&=-\nabla U(q)\, dt-\nu \frac{p}{m}\, dt-\frac{1}{m}\int _{0}^t K(t-s) p(s)\,ds\, dt+F(t)\, dt+\sqrt{2\nu \beta ^{-1}}\, d W(t). \end{aligned}$$

In the above equation, *q* and *p* denote the particle’s position and momentum; $m>0$ is the mass of the particle, *U* is a confining external potential; $\nu \ge 0$ is the viscous drag coefficient, *K*(*t*) is a memory kernel that models the delayed drag effects exerted by the fluid on the potential; *F*(*t*) is a mean zero, stationary Gaussian process with autocovariance function, which is related to the memory kernel via the Fluctuation–Dissipation theorem [31, 39, 41, 45, 56], i.e. $\mathbb {E}[F(t)F(s)]={\beta ^{-1}}K(|t-s|)$; and finally *W*(*t*) is a standard Brownian motion. The GLE (1), endowed with suitable initial conditions, describes the motion of a microparticle in a thermally fluctuating viscoelastic fluid. It has been employed in various applications such as surface diffusion and polymer dynamics, see the aforementioned monographs [5, 45] for further information on the GLE, including its derivation from a mechanical model coupled with a thermal reservoir.

In general, due to the presence of the memory kernel, Eq. (1) describes a non-Markovian process. However, when *K*(*t*) has the form of a finite sum of exponentials, viz.

2 $$\begin{aligned} K(t)=\sum _{i=1}^M \lambda _i^2 e^{-\alpha _i t}, \quad t\ge 0 \end{aligned}$$

then it has been proved that the non-Markovian GLE can be equivalently formulated as a Markovian system by introducing *M* auxiliary variables [32, 56]. More precisely, the augmented Markovian system takes the form

3a $$\begin{aligned} d q&= \frac{p}{m}\, dt , \end{aligned}$$

3b $$\begin{aligned} d p&= - \nabla _{q}U(q) \, dt -\nu \frac{p}{m}\, dt + \sum ^{M}_{k=1} \lambda _k z_{k} \, dt+\sqrt{2\nu \beta ^{-1}}\, dW_0(t),\end{aligned}$$

3c $$\begin{aligned} d z_{k}&= - \lambda _k \frac{p}{m}\, dt - \alpha _k z_{k} \, dt + \sqrt{ 2 \alpha _k \beta ^{-1} } d W_{k}(t) , \quad k=1,\dots ,M, \end{aligned}$$

where $\{z_k\}_{k=1}^M$ are augmented variables modelling the heat bath and $\{W_k\}_{k=0}^M$ are independent standard Wiener processes. The system (3), which we still refer to as the Generalized Langevin dynamics, has been studied extensively in the literature by many authors, see [17, 18, 36, 42, 44, 50] and references therein. In particular, for suitable scaling of the involved parameters, it has been proved that the GLE (3) can be cast into the framework of fast–slow dynamics. Using such a framework, one can derive the classical structure of both the underdamped and overdamped Langevin dynamics by passing to suitable limits in the scaling parameter; for details, we refer the reader to [17, 42, 44, 50]. We formally show these asymptotic limits in the following sections.

### Model Reduction for Generalized Langevin Dynamics

**Fig. 1:**
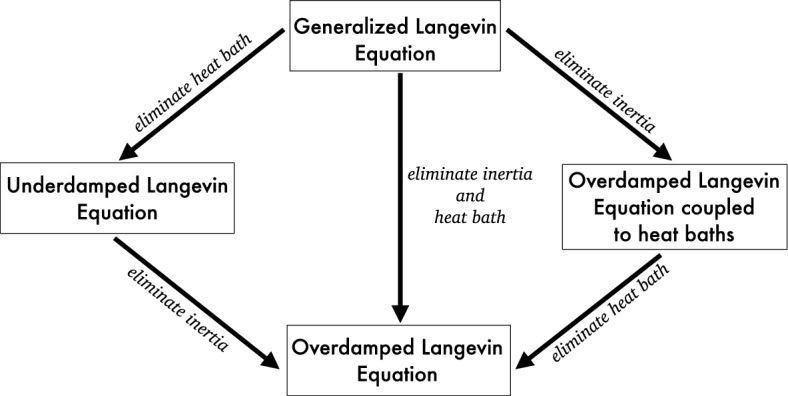
The stairs of reduction for the Generalized Langevin Equation given in Eqs. (3). The reduction procedure detailed in the text guarantees that the diagram above is commutative, under a suitable time scaling

In this paper, we carry out three different reduction paths for the GLE (3) equipped with quadratic confining potentials, in which we do not assume a priori the presence of a time-scale separation. The procedure is summarized in Fig. 1. The first path consists in deriving the underdamped Langevin dynamics described by the only position and momentum variables $\{q,p\}$ by eliminating the heat bath variables $\{z_k\}_{k=1}^M$ (see the top left arrow in Fig. 1). In the second path, we instead eliminate the inertia (i.e., the momentum variable *p*) to obtain a reduced model described by the position *q* and heat baths variables $\{z_k\}_{k=1}^M$ (top right arrow in Fig. 1). Using the same approach, we may eventually contract the description even further, by removing the inertia *p* in the first reduced model (bottom left arrow in Fig. 1) or the heat baths $\{z_k\}_{k=1}^M$ in the second one (bottom right arrow). We then show that the both reduction paths lead to the same reduced overdamped Langevin dynamics for the remaining position variable *q*. In the third path, we demonstrate that the latter reduced system can also be obtained from the GLE directly by removing both inertia and heat bath variables simultaneously (the central vertical downward arrow). In other words, the reduction diagram portrayed in Fig. 1 is commutative.

Along each step of the reduction path, we employ the same tool—the Invariant Manifold method. This constitutes the backbone of a variety of reduction procedures exploited in classical kinetic theory [20] and with Fluctuation–Dissipation relations, whose use in the set-up of model reduction was recently described in [8, 9, 13]. Thereby, starting off from a set of SDEs with additive noise and selecting a set of collective variables, a reduced model is derived in two steps: (i) the deterministic component of for the reduced dynamics is obtained using the classical Invariant Manifold method, then (ii) the diffusion terms are determined by enforcing the Fluctuation–Dissipation Theorem. For the stochastic linear systems considered in this paper, the Fluctuation–Dissipation Theorem establishes a relation between the diffusion matrix, the transport matrix, and the stationary covariance matrix, expressed by a so-called Lyapunov equation [45, 47]. We employ such relation twice, stipulating the scale invariance of the stationary covariance matrices. Thus, by construction, our approach paves the way to a multiscale connection between the transport and diffusion terms in both the original and reduced dynamics. This is the main novelty of our approach compared to existing methods in the literature. In each of the considered reduction steps, we rescale the system through a parameter $\varepsilon$, which controls the time-scale separation between the “fast” and the “slow” variables. A perfect time-scale separation is achieved in the limit $\varepsilon \rightarrow 0$, where the standard Langevin equations encountered in the literature are recovered. There are two essential advantages coming from the considered rescaling procedure. The first one is that it provides a meaningful set-up to solve the Invariance Equations. The latter, which constitute a central tool in the derivation of hydrodynamics from kinetic theory [10–12, 29, 30], quantify the following conceptual recipe: the vector field of the original (deterministic) dynamics coincides with its projection onto a manifold parameterized by a finite set of slow variables, see also [21, 23]. Introducing an expansion of the fast variables in powers of $\varepsilon$ (that hence resembles the classical Chapman–Enskog expansion known in kinetic theory of gases [6]) makes it possible to solve iteratively the Invariance Equations. The second advantage is that this procedure makes it possible to recover the aforementioned asymptotic limits as the leading order in the Chapman–Enskog expansion. Namely, our iterative solution of the Invariance Equations provides finite order corrections to the limiting dynamics, that address a perfect time-scale separation. We point out that our approach might also work without formally introducing the scaling parameter $\varepsilon$. Indeed, as discussed in [21], one could set this parameter to unity (i.e., letting $\varepsilon =1$), and in some cases it is even possible to absorb it into some properly rescaled time and space variables, see e.g. [10, 11, 21]. From this perspective, our approach is different from existing methods which require the existence of a scale-separation parameter such as the averaging principle or asymptotic expansion methods [44, 46], but is similar to those that do not require an explicit scale-separation parameter such as the conditional expectation method [33, 34]. In the following sections, for the simplicity and readability of the presentation, we provide detailed calculations for the case of one heat bath ($M=1$), but our approach is applicable to the general case of arbitrarily large, but finite, number of heat baths; see Remark 3.1 for further discussions.

## From the GLE to the Underdamped Langevin Dynamics

In this section, we formally show, first, that the underdamped Langevin dynamics can be obtained from the GLE in the limit of perfect time-scale separation, which has already been proved rigorously in [44]. Later we perform our reduction procedure by eliminating the heat bath variables $\{z_k\}_{k=1}^M$ (top left arrow in Fig. 1), thus obtaining higher order corrections to the underdamped Langevin dynamics.

### Formal $\varepsilon \rightarrow 0$ Limit

Rescaling the coefficients in (3) according to the rule $\lambda _k\rightarrow \lambda _k/\varepsilon ,~\alpha _k\rightarrow \alpha _k/\varepsilon ^2$, we obtain the following rescaled generalized Langevin dynamics

4a $$\begin{aligned} d q^\varepsilon&= \frac{p^\varepsilon }{m}\, dt , \end{aligned}$$

4b $$\begin{aligned} d p^\varepsilon&= - \nabla _{q}U(q^\varepsilon ) \, dt-\nu \frac{p^\varepsilon }{m}\, dt + \sum ^{M}_{k=1} \frac{\lambda _k}{\varepsilon } z^\varepsilon _{k} \, dt+\sqrt{2\nu \beta ^{-1}}\, dW_0(t), \end{aligned}$$

4c $$\begin{aligned} d z^\varepsilon _{k}&= - \frac{\lambda _k}{\varepsilon }\frac{p^\varepsilon }{m}\, dt - \frac{\alpha _k}{\varepsilon ^2} z^\varepsilon _{k} \, dt + \sqrt{ 2\frac{\alpha _k}{\varepsilon ^2} \beta ^{-1} } d W_{k}(t) , \quad k=1,\dots ,M , \end{aligned}$$

where, as previously stated, $\{W_k\}_{k=0}^M$ are independent standard Wiener processes.

Next, we formally derive the limiting system as $\varepsilon \rightarrow 0$ from (4); we refer the reader to [44] for a rigorous proof of this passage to the limit. From (4c), we get

$$\begin{aligned} \frac{1}{\varepsilon }z^\varepsilon _k \, dt&=\frac{\varepsilon }{\alpha _k}\Big [{-}dz^\varepsilon _k-\frac{\lambda _k}{\varepsilon }\frac{p^\varepsilon }{m}\, dt + \sqrt{ 2\frac{\alpha _k}{\varepsilon ^2} \beta ^{-1} } d W_{k}(t)\Big ] \\  &={-}\frac{\varepsilon }{\alpha _k}dz^\varepsilon _k-\frac{\lambda _k}{\alpha _k}\frac{p^\varepsilon }{m}\,dt+\sqrt{\frac{2\beta ^{-1}}{\alpha _k}}\, d W_{k}(t). \end{aligned}$$

Substituting this expression into (4b) yields

$$\begin{aligned} d p^\varepsilon&= - \nabla _{q}U(q^\varepsilon ) \, dt {-} \varepsilon \sum ^{M}_{k=1}\frac{\lambda _k}{\alpha _k}dz^\varepsilon _k-\Big (\nu +\sum _{k=1}^M\frac{\lambda _k^2}{\alpha _k}\Big )\frac{p^\varepsilon }{m}\,dt\\&\quad +\Big (\sqrt{2\nu \beta ^{-1}}\, dW_0(t)+\sum _{k=1}^M\sqrt{\frac{2\lambda _k^2\beta ^{-1}}{\alpha _k}}\, d W_{k}(t)\Big )\\&=- \nabla _{q}U(q^\varepsilon ) \, dt {-} \varepsilon \sum ^{M}_{k=1}\frac{\lambda _k}{\alpha _k}dz^\varepsilon _k -\gamma \frac{p^\varepsilon }{m}\,dt+\sqrt{2\gamma \beta ^{-1}}\, dW_t, \end{aligned}$$

where the friction coefficient $\gamma$ is defined by

5 $$\begin{aligned} \gamma = \nu +\sum ^{M}_{k=1} \frac{\lambda ^{2}_{k}}{\alpha _{k}} . \end{aligned}$$

To reach this point, we have used the fact that

$$ \sqrt{\nu }\, dW_0(t)+\sum _{k=1}^M\sqrt{\frac{\lambda _k^2}{\alpha _k}}\, d W_{k}(t) $$

is a Brownian motion with mean zero and variance $\gamma$. Hence, this can be written as $\sqrt{\gamma }W(t)$, where *W*(*t*) is the standard Brownian motion. As $\varepsilon \rightarrow 0$, it yields that the process $(q^\varepsilon , p^\varepsilon )$ converges to

6a $$\begin{aligned} d q&= \frac{p}{m}\, dt , \end{aligned}$$

6b $$\begin{aligned} d p&= - \nabla _{q}U(q) \, d t - \gamma \frac{p}{m}\, dt + \sqrt{2\gamma \beta ^{-1}} d W(t) , \end{aligned}$$

where the friction coefficient $\gamma$ is defined in (5) and *W*, again, is a standard Wiener process.

### Elimination of the Heat Bath variables

We start off now from the rescaled generalized Langevin dynamics (4). In the sequel, we denote by $\langle X \rangle :=\mathbb {E}[X]$ the expected value of the random variable *X*. We look for a reduced description of the model described by Eq. (3) subject a harmonic potential $U(x)=1/2m\omega ^2 q^2$, obtained by eliminating the set of heat bath variables $\{z_k\}_{k=1}^M$. We fix $M=1$ in Eq. (3), then rescale $\lambda \rightarrow \lambda /\varepsilon$, $\alpha \rightarrow \alpha /\varepsilon ^2$ and finally rescale also time as $t \rightarrow \varepsilon ^2 t$. Noting that $dW(\varepsilon ^2 t)=\varepsilon dW(t)$, we can rewrite the original dynamics as a linear system of SDEs:

7 $$\begin{aligned} d{\textbf{z}}=\textbf{M}_{\varepsilon } \textbf{z} dt+\varvec{{\sigma }}_{\varepsilon } d\textbf{W}(t) , \end{aligned}$$

where $$\textbf{z}=(q,p,z)^T$$, $$d\textbf{W}(t)=(0,dW_0(t),dW(t))^T$$,

8 $$\begin{aligned} \textbf{M}_{\varepsilon }= \begin{pmatrix} 0 &  \varepsilon ^2/m &  0 \\ -\varepsilon ^2 m \omega ^2 &  -\varepsilon ^2\nu /m &  \varepsilon \lambda \\ 0 &  -\varepsilon \lambda /m &  -\alpha \end{pmatrix} , \end{aligned}$$

and

9 $$\begin{aligned} \varvec{{\sigma }}_{\varepsilon }= \begin{pmatrix} 0 &  0 &  0 \\ 0 &  \varepsilon \sqrt{2 \nu /\beta } &  0 \\ 0 &  0 &  \sqrt{2 \alpha /\beta } \end{pmatrix} . \end{aligned}$$

We target a reduced dynamics possessing the structure

10 $$\begin{aligned} d\textbf{x}= \textbf{M}_{r}(\varepsilon ) \textbf{x}\, dt + \varvec{{\sigma }}_{r}(\varepsilon )\, d\textbf{W}_r(t) , \end{aligned}$$

with $\textbf{x}=(q,p)^T$ and $d\textbf{W}_r(t)=(dW_0(t),dW(t))^T$. The $2\times 2$ matrices $\textbf{M}_{r}$ and $\varvec{{\sigma }}_{r}$, whose dependence on $\varepsilon$ is explicitly indicated in Eq. (10), will be determined exploiting the Invariant Manifold method and the Fluctuation–Dissipation Theorem (FDT). We shall address the corresponding derivation in the coming sections.

### Invariance Equations and the Chapman–Enskog Expansion

Profiting from the linearity of the system (8), we seek a closure relation of the form:

11 $$\begin{aligned} \langle z \rangle= &   a(\varepsilon ) \langle q \rangle + b(\varepsilon ) \langle p \rangle , \end{aligned}$$

where *a*, *b* are regarded as functions of $\varepsilon$ (the other parameters of the model are considered as fixed). One can then compute the time derivative of $\langle z \rangle$ in two different ways; see [8, 13, 20] for similar arguments. In the first, one uses directly the bottom row of (8), relying also on the closure (11). Alternatively, one considers the evolution of the fast variable $\langle z \rangle$ as driven by the slow variables $\langle q \rangle$ and $\langle p \rangle$. This way, the chain rule applies and, via (11), this gives $\langle \dot{z}\rangle =a(\varepsilon ) \, \langle \dot{q}\rangle + b(\varepsilon ) \, \langle \dot{p}\rangle$, where the time derivative of the slow variables is picked from the top and central rows of (8). Within the framework of Invariant Manifold the two foregoing expressions of the time derivative of $\langle z \rangle$ coincide, which thus leads to two *Invariance Equations* to be solved for the unknown functions $a(\varepsilon ),b(\varepsilon )$:

12a $$\begin{aligned}  &   -\varepsilon ^2m \omega ^2 b+\varepsilon \lambda ab + \alpha a = 0 , \end{aligned}$$

12b $$\begin{aligned}  &   \varepsilon ^2 a-\varepsilon ^2 \nu b +\varepsilon \lambda m b^2+ \varepsilon \lambda +\alpha m b = 0 . \end{aligned}$$

The closure relation (11) implies that

13 $$\begin{aligned} \textbf{M}_{r}(\varepsilon )=\varepsilon ^2\begin{pmatrix} 0 &  1/m \\ -\Omega ^2(\varepsilon ) &  -\Gamma (\varepsilon ) \end{pmatrix} , \end{aligned}$$

with

14 $$\begin{aligned} \Omega ^2(\varepsilon )= m\omega ^2-\varepsilon ^{-1}\lambda \, a(\varepsilon ) , \quad \Gamma (\varepsilon ) = \frac{\nu }{m} -\varepsilon ^{-1}\lambda \, b(\varepsilon ) , \end{aligned}$$

where $a(\varepsilon ),b(\varepsilon )$ solve the system (12a)–(12b). Next, call

15 $$\begin{aligned} \xi ^{\pm }(\varepsilon ):=-\varepsilon ^2\frac{\Gamma (\varepsilon )\pm \sqrt{\Gamma ^2(\varepsilon )^2-4 \Omega ^2(\varepsilon )/m}}{2} \end{aligned}$$

the two eigenvalues of the matrix $\textbf{M}_r$ in (13). Among the different sets of solutions $\{a(\varepsilon ), b(\varepsilon )\}$ of Eqs. (12), the relevant ones are continuous functions fulfilling the asymptotic behavior:

16 $$\begin{aligned} \lim _{\varepsilon \rightarrow 0}\xi ^{\pm }(\varepsilon )=0 . \end{aligned}$$

The condition in (16) guarantees that, out of the full set of eigenvalues of the matrix $\textbf{M}_{\varepsilon }$, the subset of eigenvalues retained in the reduced description are just the “slow” ones, namely those which vanish as $\varepsilon \rightarrow 0$. To proceed further, we consider approximate solutions of the system (12a)–(12b) obtained through the Chapman–Enskog (CE) method. One instructive way to perform the CE procedure amounts to expanding the coefficients $a(\varepsilon ),b(\varepsilon )$ in powers of the parameter $\varepsilon$, viz.:

17 $$\begin{aligned} a(\varepsilon )=\sum _{j=0}^{\infty }\varepsilon ^j a_j , \quad b(\varepsilon )=\sum _{j=0}^{\infty }\varepsilon ^j b_j , \end{aligned}$$

with $a_j, b_j$, $j=1,\dots ,\infty$ denoting some real-valued coefficients, and by then inserting the expansions (17) into the Invariance Equations (12a)–(12b), which can thus be solved at any order of $\varepsilon$ [20, 21]. A direct calculation yields

18 $$\begin{aligned} a(\varepsilon )=o(\varepsilon ) , \quad b(\varepsilon )=-\frac{\lambda }{m\alpha }\varepsilon +o(\varepsilon ) . \end{aligned}$$

**Fig. 2:**
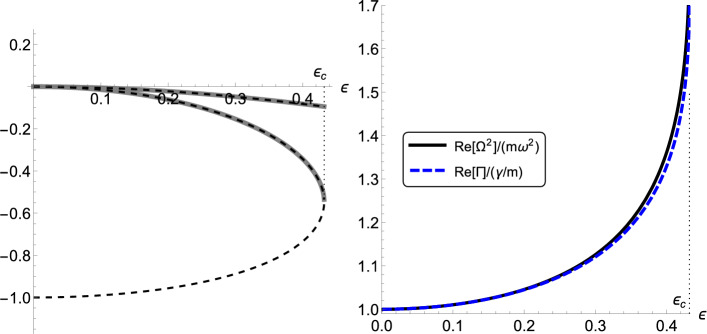
*Left panel* behavior of the real part of the eigenvalues $\xi ^{\pm }(\varepsilon )$ (thick gray lines) and of the eigenvalues of the original matrix $\textbf{M}_{\varepsilon }$ (dashed black lines), as functions of $\varepsilon$. *Right panel* behavior of the real parts of $\Omega ^2(\varepsilon )/(m\omega ^2)$ and $\Gamma (\varepsilon )/(\gamma /m)$ (solid black and blue dashed lines, respectively) defined in Eqs. (14), with the coefficients $a(\varepsilon ), b(\varepsilon )$ expressed through the solution of the Invariance Equations (12). In both panels we set $\omega =0.9$, $\lambda =\alpha =\nu =m=1$ (Color figure online)

Hence, by inserting the expressions (18) in (14), one finds

19 $$\begin{aligned} \lim _{\varepsilon \rightarrow 0} \Omega ^2(\varepsilon )=m \omega ^2 , \quad \lim _{\varepsilon \rightarrow 0} \Gamma (\varepsilon )=\frac{\nu }{m}+\frac{\lambda ^2}{m \alpha }=\frac{\gamma }{m} . \end{aligned}$$

The left panel of Fig. 2 shows the eigenvalues $\xi ^{\pm }(\varepsilon )$, which recover the eigenvalues of the matrix $\textbf{M}_{\varepsilon }$ fulfilling the requirement expressed by Eq. (16). It should be noted that an exact reduced description is available only up to a critical value $\varepsilon _c$ since, for $\varepsilon >\varepsilon _c$, two of the eigenvalues of $\textbf{M}_{\varepsilon }$ become complex conjugate. The right panel of Fig. 2 highlights the behavior of the real part of the coefficients $\Omega (\varepsilon )/(m\omega ^2)$ and $\Gamma (\varepsilon )/(\gamma /m)$, obtained through the exact solution of the Invariance Equations (12). The latter panel clearly evidences how the coefficients $\Omega ^2$ and $\Gamma$ deviate from their asymptotic limits (19) for non-vanishing values of $\varepsilon < \varepsilon _c$.

### The Fluctuation–Dissipation Theorem

Let us now turn to the computation of the matrix $\varvec{{\sigma }}_{r}$ via the FDT.

We denote by $\mathbf {\Sigma }_{\varepsilon }=\frac{1}{2}\varvec{{\sigma }}_{\varepsilon } \varvec{{\sigma }}_{\varepsilon }^T$ the diffusion matrix and by $\textbf{R}(t)$ the covariance matrix of the dynamics given in (7). One thus has20where $\textbf{R}_0(t)$ is the $2\times 2$ matrix defined as

21 $$\begin{aligned} \textbf{R}_0(t):= \left( \begin{matrix} \langle \delta q(t)^2\rangle &  \langle \delta q(t) \delta p(t) \rangle \\ \langle \delta p(t) \delta q(t) \rangle &  \langle \delta p(t)^2 \rangle \end{matrix} \right) \; , \end{aligned}$$

with $\delta q(t)=q(t)-\langle q(t)\rangle$, and an analogous notation also holds for the variables *p*(*t*) and *z*(*t*), and the stationary covariance matrix $\overline{\textbf{R}}=\lim _{t\rightarrow \infty }\textbf{R}(t)$ contains the block $\overline{\textbf{R}_0}$, corresponding to the long time limit of $\textbf{R}_0(t)$ in (21). The following relation represents an instance of the FDT [45, 47, 56], for it establishes a link between the matrices $\mathbf {\Sigma }_{\varepsilon }$, $\textbf{M}_{\varepsilon }$ and $\overline{\textbf{R}}$ in the form:

22 $$\begin{aligned} \textbf{M}_{\varepsilon }\overline{\textbf{R}}+\overline{\textbf{R}} \textbf{M}_{\varepsilon }^T=-2\mathbf {\Sigma }_{\varepsilon } . \end{aligned}$$

Analogously, we define $\overline{\textbf{R}}_r$ as the $2\times 2$ stationary covariance matrix of the reduced dynamics

23 $$\begin{aligned} \overline{\textbf{R}}_r:=\lim _{t \rightarrow \infty }\left( \begin{matrix} \langle \delta q(t)^2\rangle &  \langle \delta q(t) \delta p(t) \rangle \\ \langle \delta p(t) \delta q(t) \rangle &  \langle \delta p(t)^2 \rangle \end{matrix} \right) , \end{aligned}$$

and $\mathbf {\Sigma }_r$ as the $2\times 2$ diffusion matrix of the reduced dynamics, see Eq. (10), such that $\mathbf {\Sigma }_r=\frac{1}{2}\varvec{{\sigma }}_{r} \varvec{{\sigma }}_{r}^T$. It is possible to set-up the FDT also with the reduced dynamics, by letting:

24 $$\begin{aligned} \textbf{M}_{r}\overline{\textbf{R}_r}+\overline{\textbf{R}_r} \textbf{M}_{r}^T=-2\mathbf {\Sigma }_r . \end{aligned}$$

In order to compute $\mathbf {\Sigma }_r$ through the knowledge of $\textbf{M}_{\varepsilon }$ and $\mathbf {\Sigma }_{\varepsilon }$ we exploit an algorithm comprising the following conceptual steps: (i) we solve Eq. (22) for $\overline{\textbf{R}}$; (ii) we extract the block $\overline{\textbf{R}_0}$ from the matrix $\overline{\textbf{R}}$ and impose the scale-invariance of the correlation functions, i.e. we set $\overline{\textbf{R}_0}=\overline{\textbf{R}_r}$; (iii) we solve Eq. (24) for $\mathbf {\Sigma }_r$ [note that $\textbf{M}_{r}$ is already known from the solution of the Invariance Equations (12)]. Application of the above algorithm yields

25 $$\begin{aligned} \overline{\textbf{R}_r}= \begin{pmatrix} (m\beta \omega ^2)^{-1} &  0 \\ 0 &  m\beta ^{-1} \end{pmatrix} , \end{aligned}$$

so that the reduced diffusion matrix attains the expression

26 $$\begin{aligned} \mathbf {{\Sigma }}_{r}(\varepsilon )= \begin{pmatrix} 0 &  -\varepsilon a(\varepsilon ) \lambda /(2 \beta m \omega ^2) \\ -\varepsilon a(\varepsilon ) \lambda /(2 \beta m \omega ^2) &  -\beta ^{-1}\varepsilon [b(\varepsilon )m\lambda -\varepsilon \nu ] \end{pmatrix} . \end{aligned}$$

Upon inserting the CE expressions (18) into Eqs. (13) and (26), then rescaling time back to $t \rightarrow \varepsilon ^{-2} t$, and finally letting $\varepsilon \rightarrow 0$, one obtains

27 $$\begin{aligned} \textbf{M}_{r}(0)=\begin{pmatrix} 0 &  1/m \\ -m\omega ^2 &  -\gamma /m \end{pmatrix} \quad \text {and} \quad \varvec{{\sigma }}_{r}(0)= \begin{pmatrix} 0 &  0 \\ 0 & \sqrt{2 \gamma /\beta } \end{pmatrix} , \end{aligned}$$

that properly match the structure of Eq. (6) with $U(q)=1/2m\ \omega ^2 q^2$ and $M=1$.

#### Remark 3.1

We point out that, due to the additive structure of the heat bath variables in (3), our approach can be extended to the case where there is an arbitrarily finite number of them, that is $M>1$. We perform here the calculations for the model reduction of this section; similar calculations can be done for the model reduction steps in the subsequent sections. To this end, we use the closure

$$\begin{aligned} \langle z_k \rangle= &   a_k(\varepsilon ) \langle q \rangle + b_k(\varepsilon ) \langle p \rangle , \end{aligned}$$

where $a_k,b_k$ are yet unknown functions of the parameters $\{\omega ,\lambda _k,\alpha _k\}$ for $k=1,\ldots , M$. Thus $a_k$ and $b_k$ satisfy the Invariance Equations (12a)–(12b) where $\lambda$ and $\alpha$ are replaced by $\lambda _k$ and $\alpha _k$. The generalized coefficients $\Omega ^2$ and $\Gamma$ in (13) become

$$\begin{aligned} \Omega ^2(\varepsilon )&=m\omega ^2-\sum _{k=1}^M\frac{\lambda _k}{\varepsilon } ~a_k(\varepsilon ) , \\ \Gamma (\varepsilon )&= \frac{\nu }{m} -\sum _{k=1}^M\frac{\lambda _k}{\varepsilon } ~b_k (\varepsilon ). \end{aligned}$$

The Chapman–Enskog expansion in (18) for each *k*, $k=1,\ldots , M$, yields

$$\begin{aligned} a_k(\varepsilon )=o(\varepsilon ) , \quad b_k(\varepsilon )=-\frac{\lambda _k }{m\alpha _k}\varepsilon +o(\varepsilon ) . \end{aligned}$$

This leads to

$$ \textbf{M}_r(\varepsilon )=\varepsilon ^2\begin{pmatrix} 0& 1/m\\ -\Omega ^2(\varepsilon )& -\Gamma (\varepsilon ) \end{pmatrix}=\varepsilon ^2\begin{pmatrix} 0& 1/m\\ -m\omega ^2+\sum _{k=1}^M\frac{\lambda _k}{\varepsilon } ~a_k(\varepsilon )& -\frac{\nu }{m} +\sum _{k=1}^M\frac{\lambda _k}{\varepsilon } ~b_k (\varepsilon ) \end{pmatrix}. $$

By rescaling time as $t \rightarrow \varepsilon ^2 t$, and then passing to the limit $\varepsilon \rightarrow 0$, we obtain

$$\begin{aligned} \textbf{M}_r(0)=\begin{pmatrix} 0& 1/m\\ -m\omega ^2& -\gamma /m \end{pmatrix}. \end{aligned}$$

We perform similar computations for the stochastic terms. Consequently, we recover the underdamped Langevin equation (6) for the case $M>1$.

## From the Underdamped to the Overdamped Langevin Dynamics

In this section, we first formally show that the overdamped Langevin dynamics can be obtained from the underdamped Langevin dynamics (6) through a proper scaling. We shall, then, carry out the proposed model reduction procedure by eliminating the inertial variable (see the arrow shown in the bottom left corner of Fig. 1). To this aim, we start letting $\gamma \mapsto \gamma /\varepsilon$ and $t\mapsto t/\varepsilon$ in Eqs. (6), so as to obtain the following rescaled underdamped Langevin equation:

28a $$\begin{aligned} d q^\varepsilon&= \frac{p^\varepsilon }{\varepsilon m}\, dt , \end{aligned}$$

28b $$\begin{aligned} d p^\varepsilon&= - \frac{1}{\varepsilon }\nabla _{q}U(q^\varepsilon ) \, d t - \frac{\gamma }{\varepsilon ^2} \frac{p^\varepsilon }{m}\, dt +\frac{1}{\varepsilon }\sqrt{\frac{2\gamma }{\beta }} d W(t) . \end{aligned}$$

### Formal $\varepsilon \rightarrow 0$ Limit

We can formally pass to the limit $\varepsilon \rightarrow 0$ in the system above. From (28a) and(28b) we have

$$ dq^\varepsilon =\frac{p^\varepsilon }{\varepsilon m}\,dt=-\frac{\varepsilon }{\gamma } dp^\varepsilon -\frac{1}{\gamma }\nabla _q U(q^\varepsilon )\,dt+\sqrt{\frac{2}{\gamma \beta }}\, dW(t). $$

Letting $\varepsilon \rightarrow 0$, we obtain the overdamped Langevin dynamics

29 $$\begin{aligned} dq=-\frac{1}{\gamma }\nabla _q U(q)\,dt +\sqrt{\frac{2}{\gamma \beta }}\, dW(t) . \end{aligned}$$

### Elimination of Inertial Terms

We dwell in more detail, here, with the classical reduction step leading to the overdamped Langevin equation (29) starting from Eq. (28) with $U(q)=1/2 m \omega ^2 q^2$, by eliminating the inertial terms, cf. [13, 46]. We start from the rescaled equations (28), and rescale also time as $t \rightarrow \varepsilon ^2 t$. In the sequel, we shall retain the notation of the previous section, as long as no ambiguity arises. The starting dynamics is described by an equation sharing the structure of Eq. (7), where we now set $\textbf{z}=(q,p)^T$, $d\textbf{W}(t)=(0,dW(t))^T$, and with

30 $$\begin{aligned} \textbf{M}_{\varepsilon }=\begin{pmatrix} 0 &  \varepsilon /m \\ -\varepsilon m\omega ^2 &  -\gamma /m \end{pmatrix} , \quad \varvec{{\sigma }}_{\varepsilon }= \begin{pmatrix} 0 &  0 \\ 0 &  \sqrt{2 \gamma \beta ^{-1}} \\ \end{pmatrix} . \end{aligned}$$

We target an overdamped equation written in the form

31 $$\begin{aligned} dq = M_r(\varepsilon ) dt + \sqrt{2 \Sigma _r(\varepsilon )}\ dW(t) , \end{aligned}$$

with yet unknown functions $M_r(\varepsilon ),\Sigma _r(\varepsilon )$. We stipulate a linear closure relation $\langle p \rangle = c(\varepsilon ) \langle q \rangle$, which gives $M_r(\varepsilon )=\varepsilon c(\varepsilon )/m$. The Invariance Equation hence reads

32 $$\begin{aligned} \varepsilon c^2 + \gamma c+ \varepsilon m^2\omega ^2=0 , \end{aligned}$$

and its only relevant solution corresponds to the root of the quadratic equation (32) which vanishes for $\varepsilon \rightarrow 0$. The FDT leads to the expression $\Sigma _r(\varepsilon )=\varepsilon ^3 c(\varepsilon )/(\beta m^2 \omega ^2)$, whereas the application of the CE expansion yields:

33 $$\begin{aligned} c(\varepsilon )=-\varepsilon m^2 \omega ^2 \gamma ^{-1}+O(\varepsilon ^2) . \end{aligned}$$

Inserting (33) in the expressions of $M_r(\varepsilon )$ and $\Sigma _r(\varepsilon )$, then rescaling time back as $t \rightarrow \varepsilon ^{-2} t$ and finally letting $\varepsilon \rightarrow 0$, one obtains $M_r(0)=-m \omega ^2/\gamma$ and $\Sigma _r(0)=2(\gamma \beta )^{-1}$, thus reproducing the structure of Eq. (29).

## From the Generalized to the Overdamped Langevin Dynamics

In this section, we formally show, first, that the overdamped Langevin dynamics can be obtained *directly* from the GLE via an appropriate rescaling of the parameters. Then, we perform our reduction procedure by erasing the momentum *p* and the heat bath variables $\{z_k\}_{k=1}^M$ simultaneously (see the central vertical downward arrow in Fig. 1).

### Formal $\varepsilon \rightarrow 0$ Limit

Consider the following rescaled generalized Langevin dynamics, obtained from (3) by rescaling $t\rightarrow t/\varepsilon ^2$ and $\nu \rightarrow \nu /\varepsilon ^2$ in the position and momentum variables, as well as the coefficients $\lambda _k\rightarrow \lambda _k/\varepsilon , \alpha _k\rightarrow \alpha _k/\varepsilon ^2$:

34a $$\begin{aligned} d q^\varepsilon&= \frac{p^\varepsilon }{m \varepsilon ^2 }\, dt , \end{aligned}$$

34b $$\begin{aligned} d p^\varepsilon&= - \frac{1}{\varepsilon ^2}\nabla _{q}U(q^\varepsilon ) \, dt-\nu \frac{p^\varepsilon }{m \varepsilon ^4}\, dt + \sum ^{M}_{k=1} \frac{\lambda _k}{\varepsilon ^3} z^\varepsilon _{k} \, dt+\frac{1}{\varepsilon ^2}\sqrt{2\nu \beta ^{-1}}\, dW_0(t), \end{aligned}$$

34c $$\begin{aligned} d z^\varepsilon _{k}&= - \frac{\lambda _k}{m\varepsilon ^3}p^\varepsilon \, dt - \frac{\alpha _k}{\varepsilon ^2} z^\varepsilon _{k} \, dt + \sqrt{ 2\frac{\alpha _k}{\varepsilon ^2} \beta ^{-1} } d W_{k}(t) , \quad k=1,\dots ,M . \end{aligned}$$

We now formally derive the limiting system as $\varepsilon \rightarrow 0$. We have

$$ \frac{z^\varepsilon _k}{\varepsilon ^3}\,dt\overset{\hbox {(34c)}}{=}\Big [{-}\frac{1}{\alpha _k \varepsilon }dz^\varepsilon _k-\frac{\lambda _k}{m\alpha _k \varepsilon ^4}p^\varepsilon \,dt+\sqrt{\frac{2\beta ^{-1}}{\alpha _k}}\frac{1}{\varepsilon ^2}dW_k\Big ]. $$

Substituting this into (34b)

$$\begin{aligned} dp^\varepsilon&= - \frac{1}{\varepsilon ^2}\nabla _{q}U(q^\varepsilon ) \, dt {-} \sum ^{M}_{k=1}\frac{\lambda _k}{\alpha _k \varepsilon }dz^\varepsilon _k-\Big (\nu +\sum _{k=1}^M\frac{\lambda _k^2}{\alpha _k}\Big )\frac{p^\varepsilon }{m \varepsilon ^4}\,dt\\&\quad +\sqrt{2\nu \beta ^{-1}}\frac{1}{\varepsilon ^2}\, dW_0(t)+\sum _{k=1}^M \sqrt{\frac{2\beta ^{-1}\lambda _k^2}{\alpha _k}}\frac{1}{\varepsilon ^2}dW_k\\&=- \frac{1}{\varepsilon ^2}\nabla _{q}U(q^\varepsilon ) \, dt {-} \sum ^{M}_{k=1}\frac{\lambda _k}{\alpha _k \varepsilon }dz^\varepsilon _k-\gamma \frac{p^\varepsilon }{ m\varepsilon ^4}\,dt+\frac{1}{\varepsilon ^2}\sqrt{2\gamma \beta ^{-1}}dW(t), \end{aligned}$$

where $\gamma$ is defined in (5) and *W*(*t*) is a standard Wiener process. It follows that

$$ \frac{p^\varepsilon }{m\varepsilon ^2}\, dt=\frac{\varepsilon ^2}{\gamma }dp^\varepsilon -\frac{1}{\gamma }\nabla _q U(q^\varepsilon )\,dt{-}\frac{\varepsilon }{\gamma }\sum _{k=1}^M\frac{\lambda _k}{\alpha _k}dz^\varepsilon +\sqrt{\frac{2\beta ^{-1}}{\gamma }}\,dW(t). $$

Substituting into (34a) yields

$$\begin{aligned} d q^\varepsilon =\frac{\varepsilon ^2}{\gamma }dp^\varepsilon -\frac{1}{\gamma }\nabla _q U(q^\varepsilon )\,dt{-}\frac{\varepsilon }{\gamma }\sum _{k=1}^M\frac{\lambda _k}{\alpha _k}dz^\varepsilon +\sqrt{\frac{2\beta ^{-1}}{\gamma }}\,dW(t). \end{aligned}$$

Thus as $\varepsilon \rightarrow 0$, the process $q^\varepsilon$ converges to

35 $$\begin{aligned} dq=-\frac{1}{\gamma }\nabla _q U(q)\,dt+\sqrt{\frac{2\beta ^{-1}}{\gamma }}dW(t) , \end{aligned}$$

which is precisely the overdamped Langevin dynamics (29).

### Elimination of Inertial Terms and Heat Bath Variables

In Eqs. (34) we fix again $M=1$ and $U(q)=1/2 m \omega ^2 q^2$. Upon rescaling time as $t\rightarrow \varepsilon ^4 t$, we can rewrite the original dynamics in the form expressed by Eq. (7), where we set $\textbf{z}=(q,p,z)^T$, $d\textbf{W}(t)=(0,dW_0(t),dW(t))^T$,

36 $$\begin{aligned} \textbf{M}_{\varepsilon }= \begin{pmatrix} 0 &  \varepsilon ^2/m &  0 \\ -\varepsilon ^2 m \omega ^2 &  -\nu /m &  \varepsilon \lambda \\ 0 &  -\varepsilon \lambda /m &  -\varepsilon ^2 \alpha \end{pmatrix} , \end{aligned}$$

and

37 $$\begin{aligned} \varvec{{\sigma }}_{\varepsilon }= \begin{pmatrix} 0 &  0 &  0 \\ 0 &  \sqrt{2 \nu /\beta } &  0 \\ 0 &  0 &  \varepsilon \sqrt{2 \alpha /\beta } \end{pmatrix} . \end{aligned}$$

Note that in (37) we have exploited the scaling $dW(\varepsilon ^4 t)=\varepsilon ^{2}dW(t)$. We seek an overdamped dynamics in the form expressed by Eq. (31). We thus introduce the closure relations:

$$\begin{aligned} \langle p \rangle = a(\varepsilon ) \langle q \rangle , \; \langle z \rangle = b(\varepsilon ) \langle q \rangle , \end{aligned}$$

which lead to the expression $M_r(\varepsilon )=\varepsilon ^2 a(\varepsilon )/m$ and, via the FDT, also to $\Sigma _r(\varepsilon )=-\varepsilon ^2 a(\varepsilon )/(\beta m^2 \omega ^2)$. The functions $a(\varepsilon ),b(\varepsilon )$ are obtained as solutions of the Invariance Equations:

38a $$\begin{aligned}  &   \varepsilon ^2 m^2 \omega ^2+\nu a -\varepsilon \lambda m b +\varepsilon ^2 a^2 =0 , \end{aligned}$$

38b $$\begin{aligned}  &   \lambda a +\varepsilon m \alpha b+ \varepsilon a b = 0 . \end{aligned}$$

Among the different sets of solutions $\{a(\varepsilon ),b(\varepsilon )\}$ of Eqs. (38), the relevant ones are those which let $M_r$ vanish as $\varepsilon \rightarrow 0$. The application of the CE expansion yields:

39 $$\begin{aligned} a(\varepsilon )=-\varepsilon ^2 m^2 \omega ^2 \gamma ^{-1}+o(\varepsilon ^2), \quad b(\varepsilon )=\varepsilon m \omega ^2 \lambda \alpha \gamma ^{-1}+o(\varepsilon ) . \end{aligned}$$

Inserting the expansions (39) in the expressions of $M_r(\varepsilon )$ and $\Sigma _r(\varepsilon )$, then rescaling time back as $t \rightarrow \varepsilon ^4 t$ and finally letting $\varepsilon \rightarrow 0$, one gets $M_r(0)=-m \omega ^2/\gamma$ and $\Sigma _r(0)=(\gamma \beta )^{-1}$, hence recovering the structure of Eq. (29), and also matching the overdamped equation previously obtained in Sect. 4.2.

## From the Generalized Langevin to the Overdamped Langevin Dynamics Coupled to Heat Baths

In this section we consider the reduction path portrayed in the upper right corner of Fig. 1, namely we eliminate the momentum variable from the GLE. As in the previous sections, we first show that, under an appropriate scaling, one can derive a limiting dynamics that couples the position variable to the heat bath variables. Next, we shall carry out in detail our reduction procedure.

### Formal $\varepsilon \rightarrow 0$ Limit

We consider the following rescaled GLE equation obtained from (3) by rescaling $t\rightarrow t/\varepsilon$ in the position and momentum variables and $\nu \rightarrow \nu /\varepsilon$:

40a $$\begin{aligned} d q^\varepsilon&= \frac{p^\varepsilon }{\varepsilon m}\, dt , \end{aligned}$$

40b $$\begin{aligned} d p^\varepsilon&= -\frac{1}{\varepsilon } \nabla _{q}U(q^\varepsilon ) \, dt -\nu \frac{p^\varepsilon }{m\varepsilon ^2}\, dt + \frac{1}{\varepsilon }\sum ^{M}_{k=1} \lambda _k z^\varepsilon _{k} \, dt+\frac{\sqrt{2\beta ^{-1}\nu }}{\varepsilon }\, dW_0(t),\end{aligned}$$

40c $$\begin{aligned} d z^\varepsilon _{k}&= - \lambda _k \frac{p^\varepsilon }{\varepsilon m}\, dt - \alpha _k z^\varepsilon _{k} \, dt + \sqrt{ 2 \alpha _k \beta ^{-1} } d W_{k}(t) , \quad k=1,\dots ,M . \end{aligned}$$

We define

$$ y^\varepsilon _k:=\lambda _kq^\varepsilon + z^\varepsilon _k, \quad k=1,\ldots , M. $$

Then, it follows from (40a) and (40c) that

$$\begin{aligned} dy^\varepsilon _k&= - \alpha _k z^\varepsilon _{k} \, dt + \sqrt{ 2 \alpha _k \beta ^{-1} } d W_{k}(t) \\  &=\alpha _k\lambda _k q^\varepsilon \, dt-\alpha _k y^\varepsilon _k\, dt + \sqrt{ 2 \alpha _k \beta ^{-1} } d W_{k}(t). \end{aligned}$$

The GLE system (40) is equivalent to the following system in terms of $(q^\varepsilon , p^\varepsilon , y^\varepsilon )$

41a $$\begin{aligned} d q^\varepsilon&= \frac{p^\varepsilon }{\varepsilon m}\, dt , \end{aligned}$$

41b $$\begin{aligned} d p^\varepsilon&= -\frac{1}{\varepsilon } \nabla _{q}U(q^\varepsilon ) \, dt -\nu \frac{p^\varepsilon }{m\varepsilon ^2}\, dt + \frac{1}{\varepsilon }\sum ^{M}_{k=1} \lambda _k (y^\varepsilon _k-\lambda _kq^\varepsilon ) \, dt+\frac{\sqrt{2\beta ^{-1}\nu }}{\varepsilon }\, dW_0(t),\end{aligned}$$

41c $$\begin{aligned} dy^\varepsilon _k&=\alpha _k\lambda _k q^\varepsilon \, dt-\alpha _k y^\varepsilon _k\, dt + \sqrt{ 2 \alpha _k \beta ^{-1} } d W_{k}(t). \end{aligned}$$

We perform the reduction of this system by eliminating the inertia variable $p^\varepsilon$. We formally derive the limiting system by letting $\varepsilon \rightarrow 0$. From (4a) and (4b) we have

$$\begin{aligned} d q^\varepsilon = \frac{p^\varepsilon }{\varepsilon m}\, dt&=\frac{\varepsilon }{\nu }\Big (-dp^\varepsilon -\frac{1}{\varepsilon } \nabla _{q}U(q^\varepsilon ) \, dt \!+ \!\frac{1}{\varepsilon }\sum ^{M}_{k\!=\!1} \lambda _k (y^\varepsilon _k\!-\!\lambda _kq^\varepsilon ) \, dt\!+\!\frac{\sqrt{2\beta ^{-1}\nu }}{\varepsilon }\, \!dW_0(t)\Big )\\&=-\frac{\varepsilon }{\nu }dp^\varepsilon \!-\!\frac{\nabla _q U(q^\varepsilon )}{\nu }\, dt+\frac{1}{\nu }\sum _{k=1}^M \lambda _k (y^\varepsilon _k-\lambda _kq^\varepsilon )\,dt+\sqrt{\frac{2\beta ^{-1}}{\nu }}\, dW_0(t). \end{aligned}$$

By sending $\varepsilon \rightarrow 0$, we obtain a coupled system involving *q* and $y_k$

42a $$\begin{aligned} d q&= -\frac{1}{\nu }\Big (\nabla _q U(q)+\sum _{k=1}^M\lambda _k^2 q\Big )\, dt+\frac{1}{\nu }\sum _{k=1}^M \lambda _k y_k\,dt +\sqrt{\frac{2\beta ^{-1}}{\nu }}\, dW_0(t),\end{aligned}$$

42b $$\begin{aligned} dy_k&=\alpha _k\lambda _k q \, dt - \alpha _k y_{k}\,dt + \sqrt{ 2 \alpha _k \beta ^{-1} } d W_{k}(t) , \quad k=1,\dots ,M . \end{aligned}$$

Let

$$ \bar{U}(q):=U(q)+\frac{1}{2}\sum _{k=1}^M \lambda _k^2 q^2 $$

denote the effective potential. Then the limiting system can be written as

43a $$\begin{aligned} d q&= -\frac{1}{\nu }\nabla _q \bar{U}(q)\, dt+\frac{1}{\nu }\sum _{k=1}^M \lambda _k y_k\,dt+\sqrt{\frac{2\beta ^{-1}}{\nu }}\, dW_0(t), \end{aligned}$$

43b $$\begin{aligned} dy_k&=\alpha _k\lambda _k q \, dt - \alpha _k y_{k}\,dt + \sqrt{ 2 \alpha _k \beta ^{-1} } d W_{k}(t) , \quad k=1,\dots ,M . \end{aligned}$$

This system directly couples the position variable *q*, characterizing an overdamped Langevin dynamics, to the heat bath variables. This kind of system has been studied by many authors in the literature in the context of dynamical systems driven by colored noise, see e.g. [25]. Rigorous analysis for the above asymptotic limit has also been done in the literature recently [17].

In particular, when $U(q)=1/2 m\omega ^2 q^2$, the above system boils down to

44a $$\begin{aligned} d q&= -\frac{1}{\nu }\Big (m\omega ^2+\sum _{k=1}^M\lambda _k^2\Big )q\, dt+\frac{1}{\nu }\sum _{k=1}^M \lambda _k y_k\,dt+\sqrt{\frac{2\beta ^{-1}}{\nu }}\, dW_0(t), \end{aligned}$$

44b $$\begin{aligned} dy_k&=\alpha _k\lambda _k q \, dt - \alpha _k y_{k}\,dt + \sqrt{ 2 \alpha _k \beta ^{-1} } d W_{k}(t) , \quad k=1,\dots ,M . \end{aligned}$$

Let

$$ \zeta _0:=\frac{1}{\nu }\Big (m\omega ^2+\sum _{k=1}^M\lambda _k^2\Big ),\quad \eta _k:=\frac{1}{\nu }\lambda _k, \quad \zeta _k:=\alpha _k \lambda _k. $$

Then, Eq. (44) reads

45a $$\begin{aligned} d q&= - \zeta _0 q \, dt + \sum ^{M}_{k=1} \eta _k y_{k} \, dt+\sqrt{\frac{2\beta ^{-1}}{\nu }}\, dW_0(t) , \end{aligned}$$

45b $$\begin{aligned} d y_{k}&= \zeta _k q \,dt- \alpha _k y_{k} \, dt + \sqrt{ 2 \alpha _k \beta ^{-1} } d W_{k}(t) , \quad k=1,\dots ,M . \end{aligned}$$

### Elimination of Inertial Terms

We start our reduction procedure from the rescaled GLE equation (41), where we rescale time as $t\rightarrow \varepsilon ^2 t$. The resulting dynamics can thus be cast in the structure of Eq. (7), where we now set $\textbf{z}=(q,p,y)^T$, $d\textbf{W}(t)=(0,dW_0(t),dW(t))^T$,

46 $$\begin{aligned} \textbf{M}_{\varepsilon }= \begin{pmatrix} 0 &  \varepsilon /m &  0 \\ -\varepsilon (m\omega ^2{+}\lambda ^2) &  -\nu /m &  \varepsilon \lambda \\ \varepsilon ^2 \lambda \alpha &  0 &  -\varepsilon ^2\alpha \end{pmatrix} , \end{aligned}$$

and

47 $$\begin{aligned} \varvec{{\sigma }}_{\varepsilon }= \begin{pmatrix} 0 &  0 &  0 \\ 0 &  \sqrt{2 \nu /\beta } &  0 \\ 0 &  0 &  \varepsilon \sqrt{2 \alpha /\beta } \end{pmatrix} . \end{aligned}$$

We seek a reduced description described by Eq. (10), with $\textbf{x}=(q,y)^T$ and $d\textbf{W}_r(t)=(dW_0(t),dW(t))^T$. The considered closure takes here the form

$$\begin{aligned}\langle p \rangle = a(\varepsilon ) \langle q \rangle + b(\varepsilon ) \langle y \rangle \; , \end{aligned}$$

which thus yields

48 $$\begin{aligned} \textbf{M}_{r}(\varepsilon )=\begin{pmatrix} \varepsilon a(\varepsilon )/m &  \varepsilon b(\varepsilon )/m \\ \varepsilon ^2\lambda \alpha &  -\varepsilon ^2\alpha \end{pmatrix} , \end{aligned}$$

whereas application of the FDT leads to

49 $$\begin{aligned} \mathbf {\Sigma }_{r}(\varepsilon )=\begin{pmatrix} -\frac{(a(\varepsilon ) + \lambda b(\varepsilon )) \varepsilon }{m^2 \beta \omega ^2} &  -\frac{[(a(\varepsilon ) + \lambda b(\varepsilon ))\lambda + b(\varepsilon ) m \omega ^2] \varepsilon }{2m^2 \beta \omega ^2} \\ -\frac{[(a(\varepsilon ) + \lambda b(\varepsilon ))\lambda + b(\varepsilon ) m \omega ^2] \varepsilon }{2m^2 \beta \omega ^2} &  \frac{\varepsilon ^2\alpha }{\beta } \end{pmatrix} . \end{aligned}$$

The Invariance Equations read:

$$\begin{aligned}  &   \varepsilon m^2 \omega ^2+\nu a+\varepsilon m \lambda ^2+\varepsilon a^2 + \varepsilon ^2 m\alpha \lambda b = 0 , \\  &   \nu b-\varepsilon m\lambda +\varepsilon a b-\varepsilon ^2 m \alpha b = 0 , \end{aligned}$$

while CE method returns the expressions

51 $$\begin{aligned} a(\varepsilon )=-m \zeta _0 \varepsilon + o(\varepsilon ) , \; b(\varepsilon )=m\eta \varepsilon +o(\varepsilon ) . \end{aligned}$$

Inserting the expansions (51) into (48) and (49), then rescaling time back as $t \rightarrow \varepsilon ^{-2} t$ and finally letting $\varepsilon \rightarrow 0$ yields

52 $$\begin{aligned} \textbf{M}_{r}(0)=\begin{pmatrix} - \zeta _0 &  \eta \\ \zeta &  -\alpha \end{pmatrix} , \end{aligned}$$

and

53 $$\begin{aligned} \varvec{{\sigma }}_{r}(0)=\begin{pmatrix} \sqrt{2/(\beta \nu )} &  0 \\ 0 &  \sqrt{2 \alpha /\beta } \end{pmatrix} . \end{aligned}$$

The expressions (52)–(53) correctly recover the structure of Eqs. (45).

## From the Overdamped Langevin-Heat Bath Equation to the Overdamped Langevin

In this section, we carry out the last reduction step, shown in the bottom right corner of Fig. 1, namely we eliminate the heat baths in the coupled overdamped Langevin-heat bath equation derived in the previous section.

### Formal $\varepsilon \rightarrow 0$ Limit

Consider the following rescaled system obtained from (42) by rescaling $\alpha _k\rightarrow \alpha _k/\varepsilon$:

54a $$\begin{aligned} d q^\varepsilon&= -\frac{1}{\nu }\nabla _q U(q^\varepsilon )\, dt-\frac{1}{\nu }\sum _{k=1}^M \lambda _k^2q^\varepsilon \, dt + \frac{1}{\nu }\sum ^{M}_{k=1} \lambda _ky^\varepsilon _{k} \, dt+\sqrt{\frac{2\beta ^{-1}}{\nu }}\, dW_0(t), \end{aligned}$$

54b $$\begin{aligned} d y^\varepsilon _{k}&= \frac{\lambda _k \alpha _k}{\varepsilon } q^\varepsilon \,dt- \frac{\alpha _k}{\varepsilon }y^\varepsilon _{k} \, dt + \sqrt{2 \alpha _k \beta ^{-1} \varepsilon ^{-1}} d W_{k}(t) , \quad k=1,\dots ,M . \end{aligned}$$

From this system, we will derive the overdamped Langevin dynamics by letting $\varepsilon \rightarrow 0$. From (54b), we get

$$ y^\varepsilon _k\,dt=-\frac{\varepsilon }{\alpha _k} dy^\varepsilon _k+\lambda _kq^\varepsilon \,dt+\sqrt{\frac{2\beta ^{-1}\varepsilon }{\alpha _k}}\, dW_k(t). $$

Substituting this back into (54a) yields

$$\begin{aligned} d q^\varepsilon&= - \frac{1}{\nu }\Big (\nabla _q U(q^\varepsilon )+\sum _{k=1}^M\lambda _k^2(q^\varepsilon ) \, dt + \frac{1}{\nu }\sum ^{M}_{k=1} \lambda _k\Big (-\frac{\varepsilon }{\alpha _k} dy^\varepsilon _k+\lambda _k q^\varepsilon \,dt\\&\quad +\sqrt{\frac{2\beta ^{-1}\varepsilon }{\alpha _k}}\, dW_k(t)\Big )+\sqrt{\frac{2\beta ^{-1}}{\nu }}\, dW_0(t) \\  &=-\frac{1}{\nu } \nabla _q U(q^\varepsilon )\,dt-\frac{\varepsilon }{\nu }\sum ^{M}_{k=1} \frac{\lambda _k }{\alpha _k} dy^\varepsilon _k+\frac{1}{\nu }\sqrt{2\beta ^{-1}\varepsilon \sum _{k=1}^M\frac{\lambda _k^2}{\alpha _k}}\, dW_k(t)+\frac{1}{\nu }\sqrt{2\beta ^{-1}\nu }\, dW_0(t). \end{aligned}$$

Next, upon sending $\varepsilon \rightarrow 0$ we get

55 $$\begin{aligned} dq=-\frac{1}{\nu } \nabla _q U(q)\,dt+\sqrt{\frac{2\beta ^{-1}}{\nu }}\, dW(t), \end{aligned}$$

which is exactly the overdamped Langevin dynamics after an appropriate rescaling of time, as it will be seen below.

### Elimination of Heat Bath Variables

Let us thus apply our reduction procedure to the Langevin-heat bath equation, in order to obtain an overdamped Langevin dynamics. To this aim, consider the rescaled system (54). We let $U(q)=1/2 m \omega ^2 q^2$, rescale time as $t\rightarrow \varepsilon t$ and thus write the resulting dynamics in the form given by Eq. (7), in which we set $\textbf{z}=(q,y)^T$, $d\textbf{W}(t)=(dW_0(t),dW(t))^T$, and

56 $$\begin{aligned} \textbf{M}_{\varepsilon }= \begin{pmatrix} -\varepsilon m\omega ^2/\nu -\varepsilon \lambda ^2/\nu &  \varepsilon \lambda /\nu \\ \lambda \alpha &  -\alpha \end{pmatrix} \, \end{aligned}$$

and

57 $$\begin{aligned} \varvec{{\sigma }}_{\varepsilon }= \begin{pmatrix} \sqrt{\frac{2 \varepsilon }{\nu \beta }} &  0 \\ 0 &  \sqrt{\frac{2 \alpha }{\beta }} \end{pmatrix} . \end{aligned}$$

Our target is an overdamped equation sharing the structure of Eq. (31). We here exploit the closure relation $\langle y \rangle = c(\varepsilon ) \langle q \rangle$, which leads to

58 $$\begin{aligned} M_r(\varepsilon )=-\frac{\varepsilon (m\omega ^2+\lambda ^2 -c(\varepsilon )\lambda )}{\nu } , \end{aligned}$$

and, via the FDT, also to

59 $$\begin{aligned} \Sigma _{r}(\varepsilon )=\frac{\varepsilon (-c(\varepsilon ) \lambda +\lambda ^2 +m\omega ^2)}{m \beta \nu \omega ^2} . \end{aligned}$$

In this case the Invariance Equation reads:

60 $$\begin{aligned} \lambda \alpha \nu -\alpha c \nu +c\varepsilon m \omega ^2+c \varepsilon \lambda ^2 -\varepsilon c^2 \lambda = 0 , \end{aligned}$$

whereas the CE expansion yields $c(\varepsilon )=\lambda +O(\varepsilon )$. Inserting the latter expression into (58) and (59), then rescaling time back as $t \rightarrow \varepsilon ^{-1} t$ and by finally letting $\varepsilon \rightarrow 0$, one finds $M_r(0)=-m\omega ^2/\nu$ and $\Sigma _r(0)=1/(\beta \nu )$, which recover the coefficients appearing in Eq. (55). Finally, a further rescaling of time $t \rightarrow \nu /\gamma \ t$, such that $dW(\nu /\gamma \ t)=\sqrt{\nu /\gamma }\, dW(t)$, guarantees that the overdamped equations obtained in Sects. 4.2 and 5.2 are also properly matched. This hence proves the commutativity of the various reduction paths shown in the diagram of Fig. 1.

## Conclusions

We discussed the derivation of reduced Langevin dynamics, either underdamped or overdamped, with or without the coupling with heat bath variables, from a Generalized Langevin Equation, that stands as a paradigmatic model describing non-Markovian processes. In the approach presented here, we rescaled the various Langevin dynamics through a parameter $\varepsilon$, controlling the time-scale separation between slow and fast variables. Our procedure is based on the method of the Invariant Manifold, which unveils finite order corrections to the standard Langevin equations known in the literature. Remarkably, these are properly recovered, within our formalism, in the regime of perfect time-scale separation, attained in the limit $\varepsilon \rightarrow 0$. The considered reduction procedure is also underpinned by the Fluctuation–Dissipation Theorem, which is exploited at different levels of description through the assumed scale-invariance of the stationary covariance matrices. Our method thus leads to an explicit form of the diffusion terms in the reduced dynamics, which match well-known formulas reported in the literature when a perfect time-scale separation holds. Another noteworthy feature of our technique is that it proves to be independent of the chosen reduction path. That is, the same overdamped description can be obtained from the Generalized Langevin Equation either by erasing first the inertial terms and then the heat bath variables, or vice versa. This way, the diagram portrayed in Fig. 1 turns out being commutative.

Many relevant open questions still remain ahead. One is related, for instance, to the derivation of reduced descriptions, obtained from the Generalized Langevin Equations, through the perspective of response theory, following the guidelines traced e.g. in [1, 2, 14]. Another intriguing aspect concerns the analysis of Langevin dynamics coupled to heat baths of various species, that may thus evolve on different time scales. These topics will be a matter for future work.

## Data Availability

Data sharing not applicable to this article as no datasets were generated or analysed during the current study.
